# Systematics and Palaeoecology of Three New Acrocarpous Mosses from the Mid-Cretaceous of Kachin, Myanmar

**DOI:** 10.3390/plants14142124

**Published:** 2025-07-09

**Authors:** Zhen-Zhen Tan, Yi-Ming Cui, Lwin Mar Saing, Chun-Xiang Li, Ya Li

**Affiliations:** 1 Institute of Animal Science, Jiangsu Academy of Agricultural Sciences, Nanjing 210014, China; tanzhenzi2008@163.com; 2 Lushan Botanical Garden, Jiangxi Province and Chinese Academy of Sciences, Jiujiang 332900, China; ymcui@lsbg.cn; 3 Department of Botany, University of Mohnyin, Mohnyin 01111, Myanmar; lwinmarsaing1@gmail.com; 4 State Key Laboratory of Palaeobiology and Stratigraphy, Nanjing Institute of Geology and Palaeontology, Chinese Academy of Sciences, Nanjing 210008, China

**Keywords:** *Calymperites*, *Ditrichites*, Calymperaceae, Dicranales, Mesozoic, moss fossils

## Abstract

The mid-Cretaceous Kachin amber deposit from northern Myanmar is currently a promising locality for reconstructing Cretaceous bryophyte floras. However, the vast majority of bryophyte fossils reported from Kachin amber are epiphytic leafy liverworts of Porellales and pleurocarpous mosses of Hypnodendrales, while acrocarpous mosses are rarely discovered. In addition, terrestrial-to-lithophytic bryophytes have never been reported from Kachin amber. In this study, we describe three new species of acrocarpous mosses, *Calymperites proboscideus* sp. nov., *Calymperites chenianus* sp. nov., and *Ditrichites aristatus* sp. nov. (Dicranales *s.l.*), based on 34 whole plants and 11 fragments embedded in 13 pieces of Kachin amber. *Calymperites chenianus* is an epiphytic species based on the connection to a bark fragment, while the other two species are the first terrestrial-to-lithophytic bryophytes from Kachin amber, based on the attachment of rhizoids to soil or rock. *Calymperites chenianus* and *Calymperites proboscideus* probably represent stem group members of Calymperaceae. *Ditrichites aristatus* is likely a member of Ditrichaceae or Dicranaceae. These new findings provide compelling evidence for palaeoecological habitat reconstruction of acrocarpous mosses and significantly expand our understanding of the species diversity of bryophyte communities in the Cretaceous amber forest of Myanmar.

## 1. Introduction

As an ancient lineage of land plants, mosses can be dated back to the Paleozoic based on fossil evidence [1] and DNA-based divergence time estimates [2,3]. Today, mosses are the second-most diverse lineage of land plants, with over 12,700 species in more than 800 genera and 119 families [4,5]. Based on molecular phylogenetic analysis and sporophytic features, mosses can be classified into basal mosses, acrocarpous mosses (acrocarps), and pleurocarpous mosses (pleurocarps) [5,6,7]. Basal mosses comprise only about 5% of moss species but exhibit a high degree of morphological variations [7]. They include Takakiopsida, Sphagnopsida, Andreaeopsida, Andreaeobryopsida and Oedipodiopsida that lack peristomes, Polytrichopsida and Tetraphidopsida that have nematodontous peristomes, and Buxbaumiidae and Diphysciidae of Bryopsida that have arthrodontous peristomes [7]. Acrocarps and pleurocarps are derived mosses, comprising the rest of the subclasses of Bryopsida. Acrocarps have mostly terminal sporophytes, while pleurocarps usually have creeping shoots bearing sporophytes on specialized lateral branches [8].

A significant number of exquisitely preserved bryophyte fossils have been recently discovered in mid-Cretaceous Kachin amber, Myanmar [8,9,10,11,12,13,14,15,16,17,18,19,20,21,22,23]. They offer critical materials for studying bryophyte diversity and their ecological roles and contributions in terrestrial ecosystems during the late Mesozoic. The vast majority of bryophyte fossils reported so far from Kachin amber are epiphytic Porellalean leafy liverworts [9,10,11,12,13,14,15,16,17,18,19,20] and Hypnodendralean pleurocarpous mosses [8,21,22]. Scientists have discovered to date only one acrocarpous moss species, *Calymperites burmensis* Heinrichs et al., with three specimens from Kachin amber [19,23]. However, an overview of moss fossils indicates that most Cretaceous mosses are acrocarps, while pleurocarps did not become more numerous than acrocarps until the Eocene [24]. Furthermore, the bryophyte fossils reported to date from Kachin amber have been predominantly referred to as epiphytic plants, while terrestrial-to-lithophytic bryophytes growing on the forest floor have not been reported.

Recent examinations of several collections have led to the identification of 34 whole plants and 11 fragments of acrocarpous mosses embedded in 13 pieces of mid-Cretaceous Kachin amber from Myanmar. Their excellent state of preservation enabled the study of many characters of gametophytes. These new inclusions allowed for a description of *Ditrichites aristatus* sp. nov. (Dicranales *s.l.*) and provided evidence for the presence of another two new species of *Calymperites* Ignatov et Perkovsky. Some plants and rhizoids were still attached to bark, soil, or stone, which provided direct evidence for the reconstruction of their palaeoecological habitats.

## 2. Results

### Systematic Palaeobotany

Phylum: Bryophyta

Class: Bryopsida

Subclass: Dicranidae

Order: Dicranales *s.l.*

Family: Calymperaceae

Genus: *Calymperites* Ignatov et Perkovsky

Species: Calymperites proboscideus Y.Li, sp. nov.

**Figure 1:**
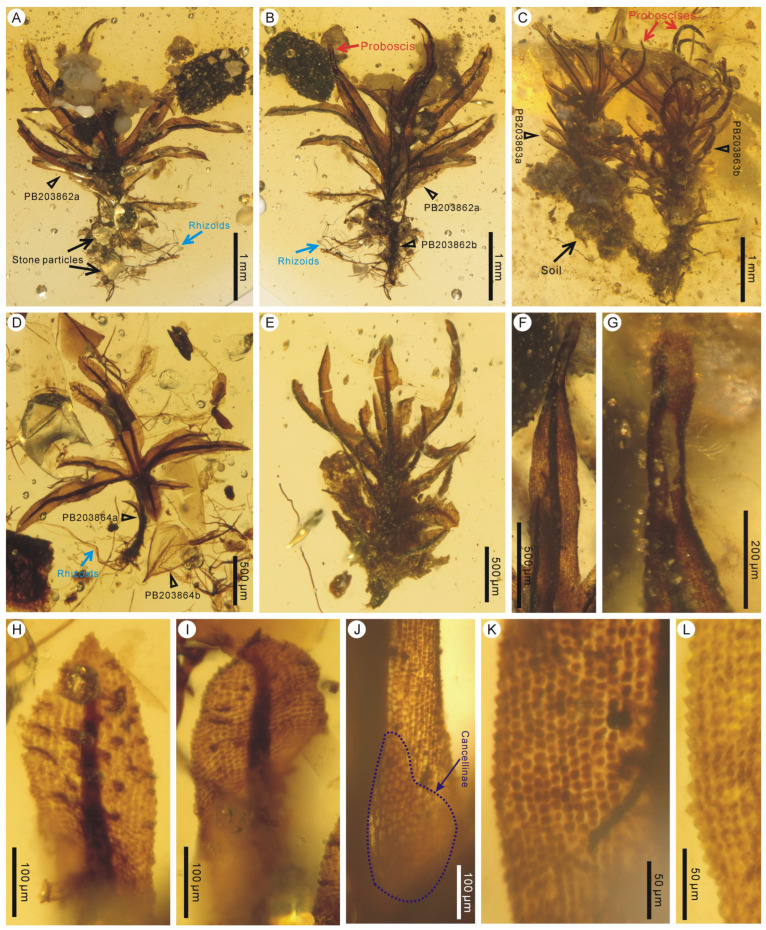
*Calymperites proboscideus* sp. nov. from mid-Cretaceous Kachin amber, Myanmar. (**A**,**B**) Tufts of moss in front and back views, showing well-developed rhizoids, a proboscis, and attached stone particles (PB203862a, b). (**C**) A tuft of moss with lower parts enclosed in soil (PB203863a, b). (**D**) A tuft of moss and an accompanying unknown moss leaf (PB203864a, b). (**E**) A moss fragment (PB203865). (**F**,**G**) Abaxial view of a leaf and its enlargement showing a short proboscis-like leaf tip (PB203862a). (**H**,**I**) Acute and mucronate leaf apices (PB203864a). (**J**) Leaf basal portion showing hyaline cells and adjacent bulging lamina cells (PB203862a). (**K**,**L**) Leaf upper portions showing finely denticulate-to-serrulate leaf margins and subquadrate, bulging lamina cells (PB203862a, PB203864a).

Holotype: PB203862a (Figure 1A,B)

**Figure 2:**
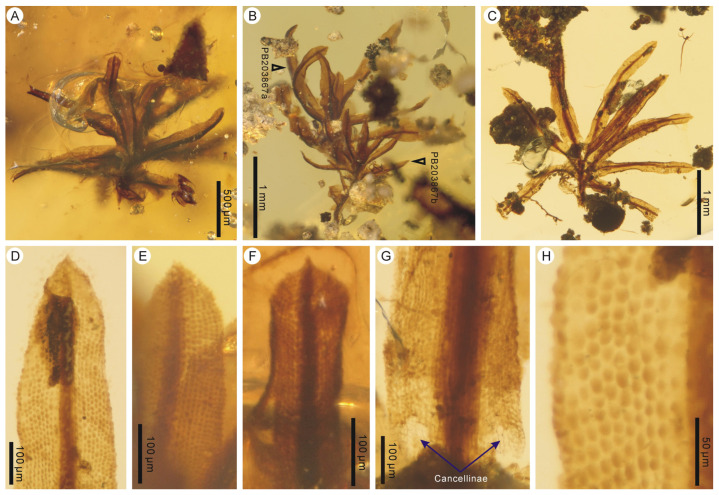
*Calymperites proboscideus* sp. nov. from mid-Cretaceous Kachin amber, Myanmar. (**A**–**C**) Detached upper parts of mosses (PB203866–PB203868). (**D**–**F**) Acute and mucronate leaf apices (PB203868, PB203867a, PB203866). (**G**) Leaf basal portion showing hyaline cells and adjacent bulging lamina cells (PB203868). (**H**) Leaf upper portion showing a finely denticulate leaf margin and subquadrate, bulging lamina cells (PB203868).

Paratypes: PB203862b, PB203863a, b, PB203864a, PB203865, PB203866, PB203867a, b, PB203868 (Figure 1 and Figure 2).

Age: Late Albian–early Cenomanian, mid-Cretaceous.

Type locality: Amber mines southwest of the village of Tanai, ca. 105 km north of Myitkyina in Kachin State, northern Myanmar.

Etymology: The specific epithet refers to the modified, proboscis-like leaf tips.

Repository: Collection Department of Nanjing Institute of Geology and Palaeontology, Chinese Academy of Sciences, Nanjing, China.

Diagnosis: Plants minute, single to tufted, with well-developed rhizoids. Leaves sometimes dimorphic, including unmodified leaves and modified leaves with proboscis-like leaf tips, probably associated with gemma production. Unmodified leaves lanceolate, with an acute-to-mucronate apex; leaf margin finely denticulate to serrulate, not bordered; leaf basal hyaline cells clearly separated from thick-walled, bulging, upper lamina cells; upper lamina cells obviously bulging.

Description: Plants minute, erect, unbranched, 1.9–3.5 mm high, single to tufted (Figure 1A–E and Figure 2A–C). Rhizoids well developed and conspicuous, attached with rocks or soil (Figure 1A–C). Leaves sometimes dimorphic, including unmodified leaves and modified leaves with proboscis-like leaf tips, probably associated with gemma production (Figure 1A–C,F,G). Unmodified leaves crowded, spirally inserted, lanceolate, ca. 0.3–2.2 mm long and 0.12–0.34 mm wide, straight or contorted, spreading or inflexed along the costa; leaf base dilated, sheathing, clasping the stem; leaf apex acute to mucronate (Figure 1H,I and Figure 2D–F); leaf margin flat to occasionally partially involute, finely denticulate to serrulate, not bordered (Figure 1H–L and Figure 2D–H); costa single, strong, being percurrent or excurrent, or ending below leaf apex (Figure 1H,I and Figure 2D–F); leaf basal hyaline cells subquadrate to rectangular, 16–36 μm × 7–17 μm, thin walled, clearly separated from adjacent bulging upper lamina cells (Figure 1J and 2G); upper lamina cells subquadrate, 8–16 μm × 6–14 μm, thick walled, opaque, obviously bulging (Figure 1K,L and Figure 2H).

Remarks: *Calymperites proboscideus* is clearly different from the coeval *Calymperites burmensis* in having acute-to-mucronate leaf apices and clearly separated groups of leaf basal hyaline cells [23]. In addition, a few triangularly protruding, large, somewhat hyaline cells are seen near the leaf apex of *Calymperites burmensis* but are not observed in *Calymperites proboscideus*. Compared with *Calymperites ucrainicus* from Eocene Rovno amber, Ukraine, the fossil species in this study mainly differs in having lanceolate leaves with finely serrulate-to-denticulate margines, while *Calymperites ucrainicus* has oblong-lingulate leaves with subentire margines [25]. Strong leaf dimorphism and specialized short proboscis-like leaf tips are also characteristic of *Calymperites proboscideus*. Similar short proboscis-like leaf tips are present in the gemmiferous leaves of several extant species of Calymperaceae from Australia, e.g., *Calymperes afzelii* Sw. and *Calymperes taitense* (Sull.) Mitt. [26]. The proboscis-like leaf tips point to the possibility that they previously carried gemmae in *Calymperites proboscideus*. Gemmae production is generally finite. After gemmae are shed, gemmiferous leaves no longer participate in asexual reproduction [26], and their presence can often only be inferred on the basis of the characteristics of the leaf apices [23]. The attachment of rhizoids to stone particles or soil (Figure 1A–C) suggests that this fossil species was lithophytic to terrestrial.

Species: *Calymperites chenianus* Y.Li, sp. nov.

Holotype: PB203869x.

Paratypes: PB203869a–w, y, z, PB203870a–d, PB203871.

Age: Late Albian–early Cenomanian, mid-Cretaceous.

Type locality: amber mines southwest of the village of Tanai, ca. 105 km north of Myitkyina in Kachin State, northern Myanmar.

Etymology: The species is named in honor of Prof. Dr. Bang-Jie Chen (1907–1970) who was the founder of Chinese bryology.

Repository: Collection Department of Nanjing Institute of Geology and Palaeontology, Chinese Academy of Sciences, Nanjing, China.

Diagnosis: Plants minute to small, in a tuft or cushion, with scanty rhizoids. Leaves lanceolate with an acuminate to aristate apex; leaf margin usually entire, occasionally with finely denticulate teeth, not bordered; leaf basal hyaline cells clearly separated from thick-walled, bulging upper lamina cells; upper lamina cells smooth, bulging to mammillose.

**Figure 3:**
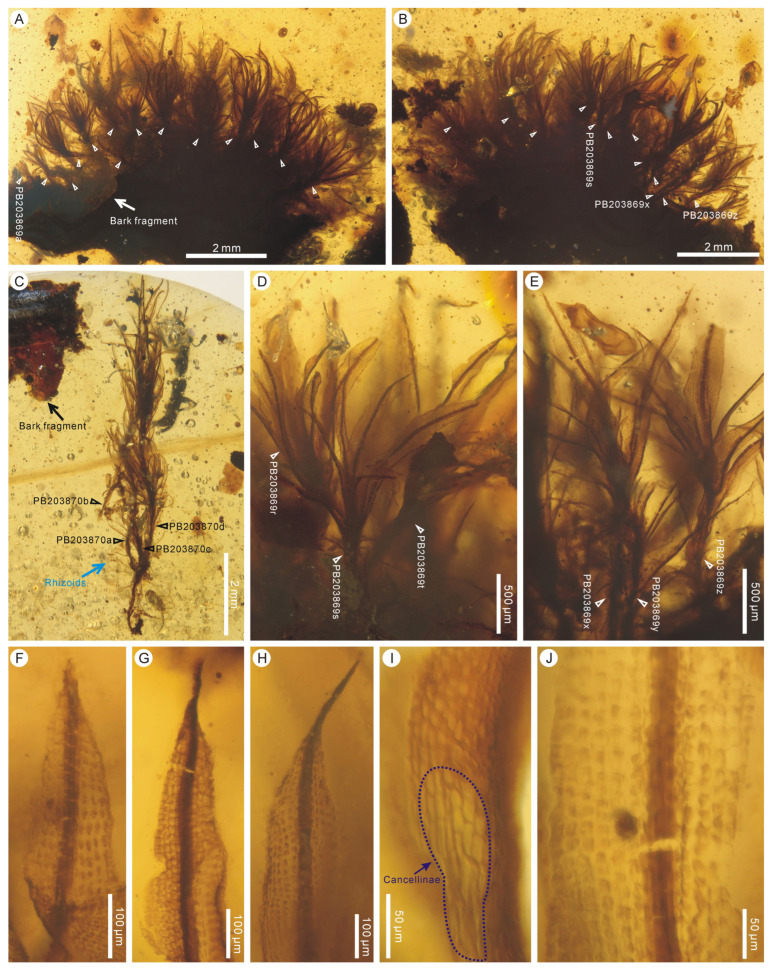
*Calymperites chenianus* sp. nov. from mid-Cretaceous Kachin amber, Myanmar. (**A**,**B**) A group of mosses (indicated by white triangles) attached to a piece of bark, in front and back views (PB203869a–z). (**C**) A tuft of mosses and the accompanying bark fragment (PB203870a–c). (**D**,**E**) Enlargements of several mosses (PB203869r–t, x–z). (**F**–**H**) Leaf upper portions showing acuminate-to-aristate apices (PB203869z, t, s). (**I**) Leaf basal portion showing hyaline cells and adjacent smooth, thick-walled lamina cells (PB203871). (**J**) Leaf upper portion showing entire margins and bulging-to-mammillose lamina cells (PB203869z).

Description: Plants minute to small, erect, unbranched, (0.8–) 1.3–3.8 (–7.3) mm high, in a tuft or cushion (ca. 26 mosses), growing on bark (Figure 3A–C). Rhizoids scanty (Figure 3C). Leaves crowded, spirally inserted, lanceolate, ca. 0.2–2.2 mm long and 0.09–0.27 mm wide, straight or contorted, spreading or inflexed along the costa (Figure 3A–E); leaf base dilated, sheathing, clasping the stem; leaf apex acuminate to shortly aristate (Figure 3F–H); leaf margin flat to occasionally partially involute, usually entire (Figure 3F,G,I,J), occasionally with finely denticulate teeth (Figure 3H), not bordered; costa single, strong, extending to the leaf apex or being excurrent (Figure 3F–H); leaf basal hyaline cells rectangular, 20–40 μm × 7–16 μm, thin walled, clearly separated from adjacent upper lamina cells (Figure 3I); upper lamina cells subquadrate to short-rectangular, 7–23 μm × 6–17 μm, thick walled, smooth, bulging to mammillose (Figure 3F–J). Gemmiferous leaves not seen.

Remarks: This fossil species is closely similar to the coeval *Calymperites burmensis* in having acuminate-to-aristate leaf apices and percurrent-to-excurrent costae but is mainly different from it in having tufted-to-gregarious habits, usually entire leaf margins, and clearly separated groups of leaf basal hyaline cells. In addition, a few triangularly protruding, large, somewhat hyaline cells are seen near the leaf apex of *Calymperites burmensis* [23] but are not observed in *Calymperites chenianus*. This fossil species differs from the coeval *Calymperites proboscideus* and the Eocene *Calymperites ucrainicus* in having acuminate-to-aristate leaf apices, while the latter two have acute-to-mucronate and obtusely acute leaf apices, respectively [25] (this paper). The attachment of mosses to bark and the accompaniment of bark fragments (Figure 3A–C) indicate that *Calymperites chenianus* is obviously an epiphytic species. Some fragments of leafy liverworts of Frullaniaceae were found inside the cushion of *Calymperites chenianus* mosses.

Order: Dicranales *s.l.*

Family: *Incertae sedis*.

Genus: *Ditrichites* Kuc.

Species: *Ditrichites aristatus* Y.Li, sp. nov.

Holotype: PB203872a.

Paratypes: PB203872b, c, PB203873, PB203874.

Age: Late Albian–early Cenomanian, mid-Cretaceous.

Type locality: Amber mines southwest of the village of Tanai, ca. 105 km north of Myitkyina in Kachin State, northern Myanmar.

Etymology: The specific epithet refers to the long, awn-like leaf apex.

Repository: Collection Department of Nanjing Institute of Geology and Palaeontology, Chinese Academy of Sciences, Nanjing, China.

Diagnosis: Plants small, single to tufted. Leaves linear-lanceolate, ca. 0.9–2.0 mm long, gradually narrowed from an ovate, sheathing base to a long aristate apex; leaf margin plane, denticulate; upper lamina cells subquadrate, rectangular to elongate-rectangular.

**Figure 4:**
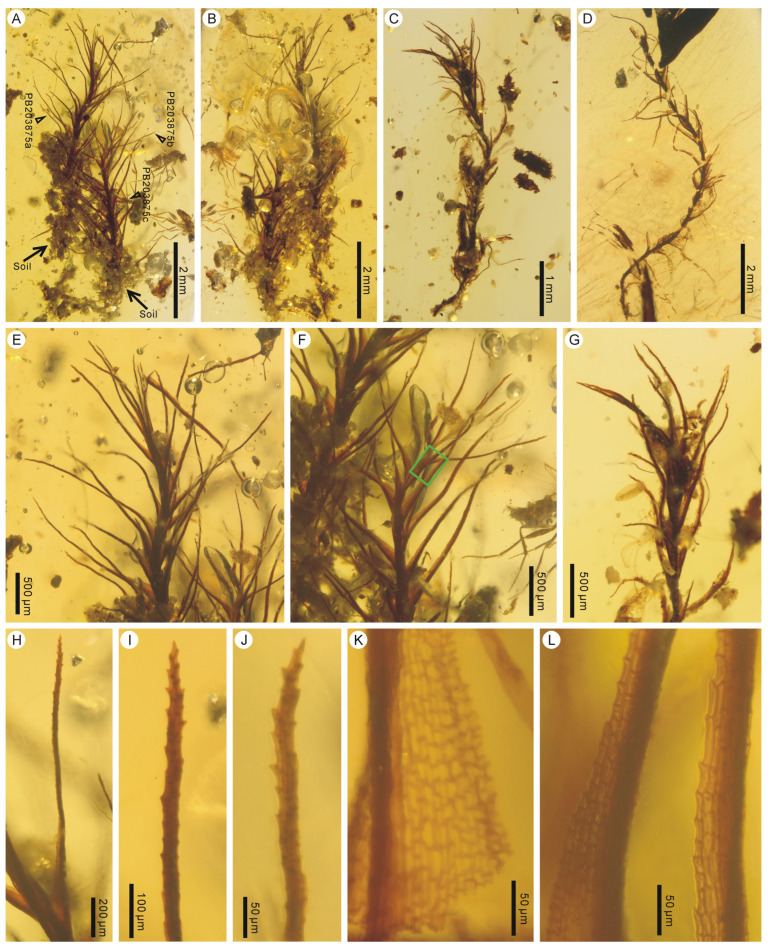
*Ditrichites aristatus* sp. nov. from mid-Cretaceous Kachin amber, Myanmar. (**A**,**B**) A tuft of moss with lower parts enclosed in soil, in front and back views (PB203872a, b, c). (**C**,**D**) Two nearly complete mosses (PB203873, PB203874). (**E**–**G**) Enlargements of upper portions of three mosses (PB203872a, b, PB203873). (**H**,**I**) A leaf and its toothed awn (PB203872a). (**J**) Another toothed awn (PB203872b). (**K**) Leaf basal portion showing subquadrate-to-rectangular cells (PB203874). (**L**) Enlargement of the green-rectangle-enclosed portion in (**F**), showing subquadrate, rectangular-to-elongate-rectangular upper lamina cells (PB203872b).

Description: Plants small, erect, unbranched, 5.4–8.7 mm high, terrestrial, single to tufted (Figure 4A–D). Lower parts of plants often attached by soil (Figure 4A–C). Leaves spirally inserted, linear-lanceolate, ca. 0.9–2.0 mm long and 0.07–0.13 mm wide, usually inflexed along the costa, gradually narrowed from an ovate, sheathing base to a long aristate apex (Figure 4E–J); leaf margin plane, denticulate; costa single, strong, long excurrent, filling most-to-entire subula (Figure 4H–J); leaf basal lamina cells subquadrate to rectangular, 11–33 μm × 6–11 μm (Figure 4K); upper lamina cells subquadrate, rectangular to elongate-rectangular, 9–49 μm × 6–10 μm, smooth (Figure 4L); alar cells not differentiated.

Remarks: *Ditrichites aristatus* is easily distinguished from the coeval fossil species of *Calymperites* by its linear-lanceolate leaves with a long aristate apex. The attachment of plants to soil (Figure 4A–C) suggests that this fossil species was terrestrial. Such leaf forms and terrestrial habit are common in Ditrichaceae [27,28] but are rare in Calymperaceae [29,30,31]. The lack of leaf basal hyaline cells excludes the placement of this species within Calymperaceae.

*Ditrichites* Kuc was established to accommodate fossil mosses that appear to be most similar to species of Ditrichaceae, especially to *Ditrichum* Hampe [32]. So far, two fossil species of *Ditrichites* have been described, including *Ditrichites fylesi* Kuc from the mid-Eocene of British Columbia, Canada [32], and *Ditrichites ignotus* J.P.Frahm from Eocene Baltic amber [33]. *Ditrichites aristatus* differs from the former species in having solid rather than tubular, cymbiform leaf upper portions and differs from the latter species in having a long aristate apex rather than a narrow lanceolate apex. In addition, *Ditrichites aristatus* is also different in having denticulate leaf margins. *Ditrichites fylesi* has entire leaf margins [32], while *Ditrichites ignotus* has entire leaf margins, occasionally with some scattered teeth in the leaf apex [33].

As the type genus of Ditrichaceae, *Ditrichum* contains ca. 90 species, distributed worldwide, and occurring from near sea level up to montane regions [27]. Molecular phylogenetic studies have indicated that *Ditrichum* is polyphyletic [34,35]. In addition, *Ditrichum* is similar to *Pleuridium* Rabenhorst (Ditrichaceae) and *Dicranella* (C.Müller) W.P.Schimper (Dicranaceae) in gametophyte morphologies and is only significantly different from *Dicranella* in its threadlike division and papillose markings of the peristome teeth [28]. Although the fossils in this study look closely similar to some extant species of *Ditrichum, Pleuridium*, and *Dicranella* [27,28,36,37], they cannot be identified to a specific family owing to the lack of sporophytes. However, despite a family-level uncertainty, the fossils in this study, along with *Calymperites* fossils, provide evidence that a diverse order Dicranales *s.l.* was already present in mid-Cretaceous Kachin amber, Myanmar.

## 3. Discussion

### 3.1. Systematic Position of Calymperites from Kachin Amber

The genus *Calymperites*, initially established for moss inclusion in Eocene Rovno amber, Ukraine [25], was later found in mid-Cretaceous Kachin amber, Myanmar [19,23]. The moss fossils of *Calymperites* from Kachin amber display several characteristics that point to Calymperaceae (Dicranales), e.g., leaf basal elongate hyaline cells forming a pair of lattices of cancellinae and the heterogeneous nature of the leaf apices [23] (this paper). Calymperaceae are a monophyletic moss family that are placed in the core Dicranales [5,38,39,40]. In the traditional classification, Calymperaceae comprise three genera, namely *Calymperes* Swartz ex F.Weber, *Mitthyridium* H.Robinson, and *Syrrhopodon* Schwägrichen, and ca. 150 species [29,30,41,42,43]. Calymperaceae are primarily distributed in tropical and subtropical regions but also have a few species ranging into temperate regions [29,44].

However, Calymperaceae have a large morphological overlap with Pottiaceae [45]. As the largest family of mosses in the number of genera, Pottiaceae are widely distributed in temperate regions of the world but are rarely found in low tropical forests [45,46]. Pottiaceae are currently classified in their own order, Pottiales [47,48], which are placed in a derived position relative to the Dicranales *s.l.* order by molecular phylogenetic studies [3,5]. *Calymperites* fossils from Kachin amber display a series of characteristics, including strong leaf dimorphism; dilated, clasping leaf bases with elongate hyaline cancellinae cells; and often toothed leaf margins. These characteristics are uniformly or commonly present in Calymperaceae but scarcely or rarely seen in Pottiaceae [45]. Furthermore, the complexly papillose distal laminal cells are an important feature of Pottiaceae [49], which are not found in *Calymperites* fossils from Kachin amber. All this evidence supports the placement of *Calymperites* from Kachin amber in Calymperaceae, which had already been proposed by Ignatov and Maslova based on leaf features of *Calymperites burmensis* [24].

Molecular dating analysis reveals that Calymperaceae diverged from their sister family around 120 Ma, with a confidence interval of 100–138 Ma [3], while their extant genera have a relatively recent origin and radiation during the Miocene [50,51]. Given the mid-Cretaceous age, *Calymperites* fossils from Kachin amber probably represent stem group members rather than a crown group of Calymperaceae, as pointed out by Bippus et al. [52]. Despite being a highly diversified moss family, Calymperaceae have a poor fossil record. *Palaeosyrrhopodon grossiseratus* Ignatov and Shcherbakov from the Early Triassic of Yaman Us, Mongolia, was initially assumed to be related to Calymperaceae based on its leaf dentition resembling that of *Syrrhopodon* [53]. But Gomankov [54] re-evaluated this fossil species by using additional Late Permian specimens from the same locality and assigned it to Isöetales (Lycopodiopsida). Unequivocal fossils of Calymperaceae have been reported from the Miocene of the Dominican Republic, including several species of the extant genera *Calymperes* and *Syrrhopodon* [55,56,57]. *Calymperites* fossils from Kachin amber represent the earliest fossils of Calymperaceae.

### 3.2. Palaeoecological Habitat Reconstruction

Epiphytic bryophytes are important components of plant biodiversity [58,59] and play important ecological roles in water and mineral cycling [60,61], as well as biological nitrogen fixation [62]. It has been suggested that epiphytic lineages are comparatively more common as fossils in amber than other lineages because the habitats of these plants were located closer to the resin flows [63]. *Calymperites chenianus* was an epiphytic moss and probably inhabited the trunks, branches, and twigs of trees. The discovery of the epiphytic moss further enriches the species diversity of the epiphytic palaeocommunities in the mid-Cretaceous Kachin amber forest, which, in addition to *Calymperites chenianus*, also includes ten leafy liverwort species belonging to *Frullania* Raddi and *Protofrullania* Heinrichs (Frullaniaceae), *Gackstroemia* Trevis. (Lepidolaenaceae) and *Radula* Dumortier (Radulaceae) [9,10,11,12,13,14,15,16,17,18,19,20], five pleurocarpous moss species of *Vetiplanaxis* Bell (Hypnodendrales) [8,21,22], and three filmy ferns of *Hymenophyllites* H.R.Goeppert and *Trichomanes* L. sensu lato (Hymenophyllaceae) [64,65].

**Figure 5:**
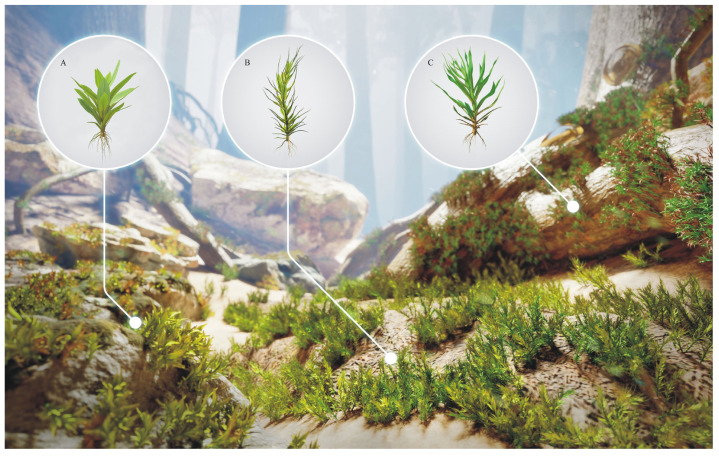
Palaeoecological habitat reconstruction of the acrocarpous moss community in the mid-Cretaceous Kachin amber forest of Myanmar. (**A**) *Calymperites proboscideus* sp. nov. (**B**) *Ditrichites aristatus* sp. nov. (**C**) *Calymperites chenianus* sp. nov.

*Ditrichites aristatus* is a terrestrial fossil species from Kachin amber. *Calymperites* displays obviously ecological niche differentiations from Kachin amber. In addition to an epiphytic species, *Calymperites* also has a terrestrial-to-lithophytic species, *Calymperites proboscideus*, which adapted to the ground and grew on soil and rocks (Figure 5). The palaeoecological habitat of *Calymperites burmensis* is still unknown. Mid-Cretaceous Kachin amber was formed under a tropical lowland forest [66,67,68], which represented the typical habitat of Calymperaceae [29,44,69]. This forest had numerous different niches and abundant micro-environmental heterogeneity, suggested by the diversity of *Frullania* (Frullaniaceae) [9,11,13,14,16,17,18] and *Vetiplanaxis* Bell (Hypnodendrales) [8,21,22], as well as the hyper-diversity of *Selaginella* (Selaginellaceae) [70,71]. Such environmental conditions favored the divergence and diversification of the moss genus *Calymperites*. Since Kachin amber from Myanmar is becoming increasingly available, further new *Calymperites* inclusions can be expected. In addition, the syn-inclusion of a detached unknown moss leaf (PB203864b in Figure 1D) with a tuft of *Calymperites proboscideus* indicates that more new bryophytes can be expected.

## 4. Materials and Methods

Kachin amber originates from several amber mines about 20 km southwest of the village of Tanai in the Hukuang Valley of Kachin State, northern Myanmar [67]. The Kachin amber deposit is currently the most important source of Cretaceous amber-preserved palaeobiota and yields a large number of plant and animal inclusions [72]. The age of Kachin amber is regarded as the late Albian–early Cenomanian, based on the evidence of the ammonite Puzosia Matsumoto and palynomorphs [66,68]. U-Pb dating of zircons suggests an earliest Cenomanian age (98.79 ± 0.62 Ma) for the amber-bearing horizon of Kachin amber [73].

Concerning the recent debates on Myanmar amber [74,75], we declare that our research followed the recommendations of Haug et al. [76]. All Kachin amber used in this study was acquired in compliance with the laws of Myanmar and China, including Myanmar’s import and export regulations of jewelry and China’s fossil law. All specimens were housed at the Collection Department of Nanjing Institute of Geology and Palaeontology, Chinese Academy of Sciences, Nanjing, China, under catalog numbers PB203862–PB203874.

The amber inclusions were observed and photographed under a ZEISS Axio Zoom.V16 microscope (Carl Zeiss AG, Oberkochen, Germany) equipped with a high-resolution digital camera (Axiocam 512 color, Carl Zeiss AG, Oberkochen, Germany). Incident light and transmitted light were used simultaneously. All images were digitally stacked as photomicrographic composites of ca. 50 individual focal planes using the software package ZEN 2.3 Pro for better illustration of the three-dimensional inclusions.

## 5. Conclusions

Here, we described three new fossil species of acrocarpous mosses, *Calymperites proboscideus* sp. nov., *Calymperites chenianus* sp. nov., and *Ditrichites aristatus* sp. nov., based on 34 whole plants and 11 detached fragments embedded in 13 pieces of mid-Cretaceous Kachin amber from Myanmar. This study raises *Calymperites* from a rare moss genus to a common genus in the mid-Cretaceous Kachin bryophyte flora and supports the placement of *Calymperites* from Kachin amber in Calymperaceae. As the earliest fossils of Calymperaceae, *Calymperites* from Kachin amber probably represents stem group members, while *Ditrichites aristatus* is likely a member of Ditrichaceae or Dicranaceae (Dicranales *s.l.*). *Calymperites* display highly ecological niche differentiations from Kachin amber, not only including an epiphytic species, but also having a terrestrial-to-lithophytic species. The findings provide convincing evidence for palaeoecological habitat reconstruction of *Calymperites* and *Ditrichites* and further enrich the species diversity of bryophyte communities in the mid-Cretaceous Kachin amber forest of Myanmar.

## Data Availability

Data are contained within the article.
